# Black Cumin (*Nigella sativa* L.) Seed Press Cake as a Novel Material for the Development of New Non-Dairy Beverage Fermented with Kefir Grains

**DOI:** 10.3390/microorganisms10020300

**Published:** 2022-01-27

**Authors:** Łukasz Łopusiewicz, Natalia Śmietana, Daria Paradowska, Emilia Drozłowska

**Affiliations:** Center of Bioimmobilisation and Innovative Packaging Materials, Faculty of Food Sciences and Fisheries, West Pomeranian University of Technology Szczecin, Janickiego 35, 71-270 Szczecin, Poland; natalia.smietana@zut.edu.pl (N.Ś.); dparadowska@gmail.com (D.P.); emilia_drozlowska@zut.edu.pl (E.D.)

**Keywords:** black cumin, *Nigella sativa* L., press cakes, dairy alternatives, fermented beverages, biotransformation, zero waste

## Abstract

In recent years, there has been a growing interest from the food industry in new products that are increasingly desired by consumers because of the functional ingredients they contain. This category certainly includes fermented plant-based beverages, which combine the properties of plant substrates with the beneficial effects of fermentation on human health. In our study, two trial variants containing 20% and 30% black cumin (*Nigella sativa* L.) seed press cake (BCPC) were inoculated with kefir grain cultures and then incubated at 25 °C for 24 h. The resulting beverages were stored under refrigeration (6 °C) for 28 days. During storage, pH, total free amino acids, reducing sugars, changes in the microbial population, viscosity, textural parameters, and color were measured on days 1, 5, 7, 14, 21, and 28. Throughout the storage period, the number of lactic acid bacteria, as well as yeasts, exceeded the recommended minimum level. Numerous changes in product parameters were observed in the tested beverages as a result of fermentation compared to non-fermented products. This study indicates the possibility of using BCPC as a valuable matrix for the production of a functional kefir-like beverage.

## 1. Introduction

New consumer behaviors have strongly affected the market for alternative dairy products. Demand for new plant-based products is driving strong growth in this sector. The need to develop modern food based on by-products of the agri-food industry is driven by consumer expectations for ecological solutions that are compatible with the circular economy concept (including European Green Deal rules). The product market share of plant-based dairy-like beverages is constantly increasing and is reaching a value of $14 billion [1]. Sources of plant-based products can be divided into several groups and the most common are cereals, legumes, nuts, seeds, and pseudo-cereals. This could also be an area for unconventional use of press cakes (the solids remaining after pressing oil from oilseeds), one of the broad groups of valuable agro-industrial by-products [2]. Previous research has outlined the potential use of press cakes as substrates for fermented plant-based dairy alternatives, such as kefir, yogurt, probiotic milk, and cheese [3,4,5]. Such products may be suitable for vegans and vegetarians as well as for people with lactose and milk protein intolerance.

*Nigella sativa* L. (black cumin) is a plant belonging to the *Ranunculaceae* family, native to southern and southwestern Asia [6]. Due to its properties, black cumin could be used in the production of functional foods (especially given its high protein and bioactive compounds). It has been used for centuries as a traditional remedy (including for asthma, hypertension, and diabetes) especially in Southeast Asia and the Middle East [7]. Black cumin seeds contain approximately 21% protein (abundant in essential amino acids such as arginine, asparagine, glutamine, cysteine, and methionine), 35% carbohydrates, and 35 to 38% fat, as well as significant amounts of bioactive compounds (such as thymoquinone), with anti-cancer, anti-inflammatory, and antibacterial properties [8,9]. The seeds significantly improve hyperglycemic parameters, reduce hypertension, and stimulate the immune system [10]. In the food industry, the seeds are often used as an additive to bread and cheese. In addition, black cumin is used for the production of paste, which is made from grounded, dehulled, and roasted seeds and is local food in the Middle East [6,10,11,12]. Black cumin seeds and their oil have been widely used in functional foods, pharmaceutical products, and nutraceuticals. Black cumin oil press cake (BCPC) is a by-product of pressing oil from whole seeds and currently is mainly used as feedstock and for conversion for bio-oil [6]; however, its potential for developing new types of functional foods is not fully exploited. 

Kefir is a fermented milk drink made from so-called kefir grains, containing bacteria and yeast. It is an easily digestible beverage and may have probiotic properties. It is an ancient drink originating from the Caucasus mountains and consists of sour fermented milk and trace amounts of alcohol [13]. Consuming kefir could satisfy the need for beneficial bacteria, yeast, vitamins, minerals, fats, and protein [14,15,16,17,18,19]. Vegan kefir substitutes should provide these compounds in adequate amounts and should be of interest to consumers. To the best of our knowledge, there are no studies on the use of BCPC for fermented beverages. Therefore, in this study, attention was given to the development of new semi-solid kefir-like products based on an unconventional matrix such as BCPC. 

## 2. Materials and Methods

### 2.1. Materials and Reagents

Black cumin oil press cake (BCPC) was kindly donated by Olejarnia Niwki (Olejarnia Niwki, Niwki, Poland). Commercial kefir grains (Yogurt-Tek^®^, Lactoferm Kefir series, Kefir-31, consisting of *Lactococcus lactis* subsp. *cremoris, Lactococcus lactis* subsp. *Lactis biotype diacetylactis, Leuconostoc mesenteroides* subsp. *cremoris, Lactobacillus delbrueckii* subsp. *Bulgaricus, and Saccharomyces cerevisiae*) were obtained from Biochem Srl (Biochem Srl, Rome, Italy). Sodium hydroxide, methanol, Folin–Ciocalteu reagent, sodium carbonate, sodium chloride, gallic acid, sodium nitrite, aluminum chloride, quercetin, 3,5-dinitrosalicylic acid, sodium tartrate tetrahydrate, ninhydrin, glacial acetic acid, cadmium chloride, and glycine were procured from Merck (Merck, Darmstadt, Germany). Glucose and hydrochloric acid were delivered by Chempur (Chempur, Piekary Śląskie, Poland). All reagents used in the presented study were of analytical purity. MRS agar (de Man, Rogosa, and Sharpe) and Sabouraud agar with chloramphenicol were received from Merck (Merck, Darmstadt, Germany).

### 2.2. Fermentation and Preparation of the Samples

The BCPC was milled and mixed with distilled water at 80 °C to obtain two concentrations (20% and 30% *w*/*w*). The resulting mixtures were boiled for 20 min with continuous stirring, and then the samples were homogenized and mixed using a home mixer Kasia Plus (MPM, Milanówek, Poland). Finally, the obtained mixtures were pasteurized for 30 min at 60 °C, then cooled down to a room temperature. Subsequently, the samples were inoculated with 10% of kefir grains (*w*/*w*) (containing 1.78 × 10^7^ ± 0.11 CFU/g of lactic acid bacteria (LAB) and 1.83 × 10^7^ ± 0.74 CFU/g of yeast). Samples obtained freshly after inoculation were used for the first analyses. The remaining kefir-like beverages were transferred to sterile low-density polyethylene cups (capacity 50 mL), which, after being tightly covered, were then incubated at 25 °C for 24 h. After incubation, the samples were stored at 5 ± 1 °C in the dark for 28 days. All analyses were performed on chosen days (0, 1, 4, 7, 14, 21 and 28).

### 2.3. Microbiological Analyses, pH Determination, and Total Solids Content (TSC)

To measure changes in pH and microbial transformations, 10 g of sample was collected and then diluted with 90 mL of sterile saline (0.9% NaCl) and serial dilutions were performed [4]. To determine the LAB, the grown colonies were cultured on MRS agar at 37 °C under anaerobic conditions for 72 h. The yeast counts were marked on Sabouraud agar supplemented with chloramphenicol at 25 °C for 72 h. Each microbial count was determined in triplicate and the number of viable cells was expressed as CFU/g of samples. To determine the pH of unfermented and fermented samples, values were detected directly at 25 °C using a pH meter (CP-411, Elmetron, Zabrze, Poland). AOAC (Association of Official Agricultural Chemists) standard method (No. 925.23) was used to evaluate the total solids content (TSC) of the samples [20]. 

### 2.4. Preparation of Extracts

The extracts of beverages were prepared as previously described [6]. The samples were freeze-dried for 24 h (chamber pressure 0.190 mbar, shelf temperature T_min_ = −35 °C, T_max_ = 20 °C, condenser temperature −85 °C) using a Beta 2-8 LSC plus lyophilizer (Martin Christ Gefriertrocknungsanlagen GmbH, Osterode am Harz, Germany). To obtain extracts, one gram of each lyophilized sample was mixed with 50 mL of solvent consisting of methanol/water solution in ratio 7:3 (*w*/*w*). Next, liquids were extracted for 15 min in an ultrasonic bath (Elmasonic S30H, Elma Schmidbauer GmbH, Singen, Germany). The samples were then centrifuged at 14,000 rpm for 10 min at 20 °C (Centrifuge 5418 Eppendorf, Warsaw, Poland) and filtered through 0.22 µm nylon membrane filters (Sigma-Aldrich, Darmstadt, Germany). The extracts obtained via this protocol were used for further spectrophotometric assays. 

### 2.5. Determination of Total Polyphenolic Content (TPC), Total Flavonoids Content (TFC), Reducing Sugars Content (RSC), and Total Free Amino Acids Level (TFAAL)

The TPC of the samples was determined using the Folin–Ciocalteu method. The 100 µL of previously prepared supernatants were mixed with 6 mL of distilled water and 0.5 mL of Folin–Ciocalteu reagent. Then 1.5 mL of saturated Na_2_CO_3_ solution was added after 3 min and the resulting mixture was incubated for 30 min in the dark at 40 °C. Absorbance was measured at 765 nm using a microplate reader (Synergy LX, BioTek, Winooski, VT, USA) by placing the samples in a 96-well microplate. TPC concentration was calculated as mg of GAE gallic acid equivalents per mL of sample (mg GAE/mL) [21].

The TFC of the samples was performed by mixing 250 µL of the supernatant with 1 mL of distilled water and 75 µL of 5% NaNO_2_ solution. After 5 min, 75 µL of 10% AlCl_3_ was added to the solution and allowed to stand for 6 min before adding 250 µL of 1 M NaOH. In the next step, distilled water was added to the mixture to make the total volume 3 mL. Absorbance was measured at 510 nm. Quercetin was used for the calibration curve and the results were expressed as mg of quercetin equivalents (QE) per mL of sample (mg QE/mL) [21].

The RSC assay was performed by the DNS (3,5-dinitrosalicylic acid) method. An appropriate amount (10 g) of DNS was dissolved in 200 mL of distilled water with continuous stirring. In the next step, the 16 g of NaOH was slowly added and dissolved in 150 mL of distilled water. The liquid was incubated at 50 °C with continuous stirring until a clear solution was obtained. Then 403 g of sodium potassium tartrate tetrahydrate was added to the obtained mixture and filtered through a paper filter (Chemland, Szczecin, Poland). The volume of the mixture was made up to 1000 mL with distilled water. In the next step, one milliliter of supernatant was combined with 1 mL of 0.05 M acetic buffer (pH 4.8) and 3 mL of DNS reagent. The whole mixture was shaken energetically and then incubated for 5 min in boiling water and then cooled at room temperature. The absorbances of the samples were measured at 540 nm (Synergy LX, BioTek, Winooski, VT, USA) and glucose in acetate buffer was used for the calibration curve [3].

The total free amino acid level (TFAAL) was determined by mixing 1 mL of supernatant with 2 mL of Cd-ninhydrin reagent, prepared following the procedure described in a previous study [4]. The mixture was vortexed and heated at 84 °C for 5 min and cooled in ice water. TFAAL was measured at absorbance 507 nm. The results obtained were expressed as milligram of Gly per mL of a sample by reference to a standard curve, which was first prepared using glycine at different concentrations.

### 2.6. Texture Analysis and Rheological Measurement

The physical assays including rheological and texture measurements of BCPC-based beverages were determined as previously described [3]. Texture profiles of the samples were performed at room temperature using a Zwick/Roell 2.5 Z apparatus (Zwick/Roell, Ulm, Germany) equipped with a cylindrical probe (diameter 40 mm). The applicated penetration rate of the samples was 10 mm/s and the depth was 25 mm. Hardness, springiness, chewiness, and gumminess were calculated from the results of the force-time curves. 

Viscosity measurements were performed in a rheometer (AR G2, TA Instruments Ltd., New Castle, DE, USA). Samples were analyzed at 20 °C with a 40 mm diameter stainless steel cone plate. Steady-state flow experiments of BCPC-based beverages were performed in the range of 0.1 to 100 s^−1^ of shear rate. The viscosity for each sample was obtained from TA Rheology Advantage Data Analysis V 5.4.7. firmware (TA Instruments, New Castle, DE, USA). Experimental flow curves were fitted to the Herschel–Bulkley (H–B) model:

$$\tau =\tau_{0}+k{\dot{\gamma }}^{n}$$

where: *τ*—the shear stress (Pa), *τ*_0_—the yield stress (Pa), $\dot{\gamma }$—the shear rate (s^−1^), ***k***—the consistency index (Pa·s^n^), *n*—the flow index [22]. 

The pseudoplastic behavior of the obtained beverages was measured using frequency oscillatory shear tests at 20 °C. The modulus G’ and G” changes were monitored at an angular frequency from 0.1 to 100 Hz and constant strain of 1%. To measure the critical strain (y_c_), strain sweep tests were performed at a strain range of 0.1–10^4^% at a constant angular frequency of 1 Hz [23]. 

### 2.7. Color Measurements

The color coordinates of the beverages were measured using a Konica Minolta CR-5 colorimeter with a Hunter Lab color system (Konica Minolta, Osaka, Japan). Results were expressed as brightness (L*), red/green (+/−a*), and yellow/blue (+/−b*). Analyses were performed at three independent times and are presented as mean values with ±standard deviation.

### 2.8. Statistical Analysis

All outcomes presented in this study were analyzed with Statistica version 10 software (StatSoft Poland, Krakow, Poland) and presented as mean ± standard deviation (SD). Statistical significance was determined using a two-way analysis of variance (two-way ANOVA) followed by Fisher’s NIR test. Differences were considered statistically significant when the *p*-value was <0.05. 

## 3. Results and Discussion

### 3.1. The Lactic Acid Bacteria (LAB) and Yeasts Viability during Storage 

**Figure 1:**
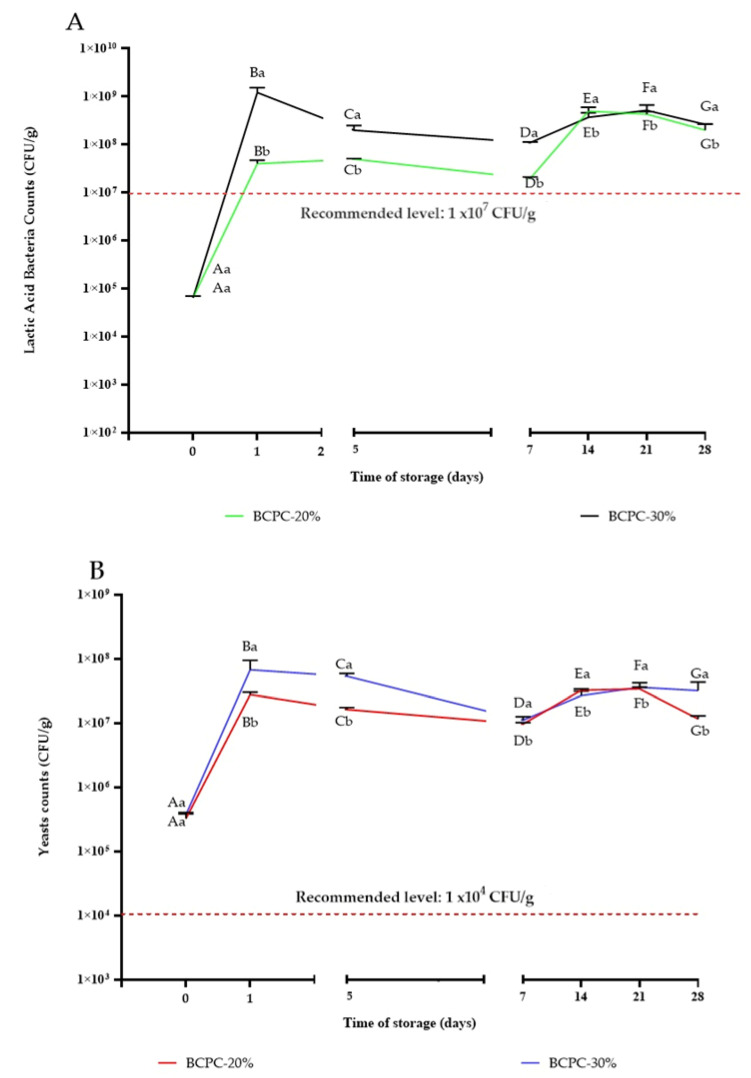
The lactic acid bacteria (**A**) and yeasts (**B**) viability during storage.

One of the important factors affecting the fermentation process is the improvement of nutritional value and sensory properties through the production of metabolites, such as organic acids (mainly lactic acid), while extending the shelf life of products by lowering the pH value, which is due to the metabolic activity of microorganisms (mainly LAB) [24,25]. The viability of microorganisms is an important factor in determining the quality of a product and has a significant impact on the colonization of the gut and the development of microbiota. As shown in Figure 1, LAB viability and yeast counts in beverages remained above recommended levels for traditional kefir i.e., >10^7^ CFU/mL and >10^4^ CFU/mL (for bacteria and yeast, respectively) [26], which is in agreement with previous reports of other authors using plant products to produce kefir-like beverages [3,18]. The highest LAB as well yeast content was noticed in sample BCPC-30% on day 1. It should be pointed out, that on day 28 the LAB and yeast contents did not fall below the recommended level for kefir. However, the BCPC is a mixed matrix of carbohydrates, proteins, residual oil, and other bioactive compounds. All compounds included in BCPC can form complexes, which could impede the accessibility of nutrients for microorganisms especially yeasts. 

It should be also remarked that BCPC could include residual oil, which remains after the cold pressing process. In a study by Arici et al. [27] five different black cumin oils were tested at three different concentrations to evaluate their potential antimicrobial properties. All tested variants of oil inhibited the activity of 16 different selected spoilage and/or pathogenic bacteria strains. The crude black cumin oil sample had higher antimicrobial activity against spoilage and pathogenic bacteria than lactic acid bacteria. However, Georgescu et al. [28] also studied the antimicrobial effect of *Nigella sativa* L. seed oil in traditionally manufactured cheese. The authors did not observe a significant reduction of LAB viability. Thus, observed effects indicated the selective antimicrobial activity of black cumin oil [28]. In our study, the number of lactic acid bacteria was not only higher than the recommended level but also no antimicrobial effect was observed during storage, indicating that in our beverage, oil residues did not inhibit the growth of kefir microorganisms.

### 3.2. pH and Reducing Sugars

Table 1 shows the pH changes during storage. On the first day, the pH levels were 5.93 ± 0.02 and 5.89 ± 0.01 (for the BCPC-20% and BCPC-30%, respectively), while on the 28th day 5.73 ± 0.03 for BCPC-20% and 5.76 ± 0.01 for BCPC-30% were recorded (*p* < 0.05). According to the literature, the pH of crude black cumin seeds is estimated at 5.63 ± 0.04 and is slightly lower than recorded in the present study [29]. It can be assumed that the BCPC matrix presumably exhibited a buffering effect during the fermentation process, given the high level of lactic acid bacteria. A similar buffering mechanism was observed by Nissen et al. in the case of the hemp seed-based fermented drinks [30]. As presented in Table 1, the RSC on day 1 was 69.19 ± 1.33 mg/g and 83.85 ± 7.42 mg/g (for the BCPC-20 and BCPC-30%, respectively), while on day 28 it was 29.12 ± 0.38 mg/g and 18.49 ± 0.19 mg/g. These results indicated the consumption of sugars by kefir microorganisms. LAB and yeasts can break disaccharides into monosaccharides by using sucrase or maltase (yeasts). This is a natural process that leads to the production of lactic acid by LAB and ethanol and acetic acid by yeasts [31]. A significant decrease in RSC was observed during fermentation (*p* < 0.05), indicating that the lactic acid bacteria and yeast have utilized the sugars for their growth. The Total Solids Content (TSC) changed during the storage time. In both samples, TSC significantly decreased after the fermentation (*p* < 0.05). During the subsequent days of cold storage, slight changes of TSC were observed. Statistically significant differences were observed on days 1, 21, and 28 (*p* < 0.05). 

### 3.3. The Changes of Total Free Amino Acid Level (TFAAL), Total Polyphenolics Content (TPC), and Total Flavonoids Content (TFC)

The changes of TFAAL are shown in Table 2. As can be seen, the TFAAL in the samples changed slightly as a result of fermentation (*p* < 0.05). On day 21 of storage, the TFAAL of the samples was 1.23 ± 0.06 mg Gly/mL for sample BCPC-20% and 1.12 ± 0.00 mg Gly/mL for BCPC-30%. On day 28, there was a significant decrease in TFAAL of the sample BCPC-20% (0.94 ± 0.02 mg Gly/mL (*p* < 0.05)). In contrast, the sample BCPC-30% showed an increase of TFAAL to 1.43 ± 0.00 mg Gly/mL. The increased TFAAL during the fermentation process might be presumably a result of the progressive proteolysis. The proteolysis effect on the TFAAL in fermented plant-based foods has been already described and allows us to put forward a thesis that the slight increase in TFAAL (especially in sample BCPC-30%) was caused by partial proteolysis. During the fermentation process, proteases are released and are breaking down the proteins of the studied matrix and changing the content of bioactive compounds in the studied samples [32]. 

As expected, the fermentation process influenced the content of bioactive compounds. The highest increase in TPC in comparison to the unfermented sample was observed on day 21 for the sample BCPC-20% and on day 28 for the sample BCPC-30% (*p* < 0.05). The TPC in the extracts of black cumin examined by Sen et al. [13] was 2.92 mg GAE/g. However, Crina-Toma et al. [33] showed that the polyphenol content of cumin extracts was 4.12 mg GAE/g. Other researchers reported that the polyphenolic content of BCPC extracts can range from 4.12 to 32.10 mg GAE per g of dry weight [34]. Obtained results were higher than presented in the cited works and increased during storage. On the other hand, a statistically significant increase in TPC could be observed only in the case of BCPC-30% on day 28 (*p* < 0.05). Analyzing the TFC of the samples, it could be observed that the highest value was obtained on day 14 for sample BCPC-30% (1.38 ± 0.15 mg GAE/mL). In their study, Ahmed et al. [35] focused on the total content of flavonoid compounds in *Nigella sativa* L. seed methanolic extracts. The authors demonstrated that the TFC in the samples ranged from 31.47 ± 1.85 mg RE/g (rutin equivalents) of the crude extract to 140.11 ± 5.47 mg RE/g of extract containing 20% ethanol. They showed that quercetin and kaempferol are major flavonoids, which could be found in black cumin seed extracts. These compounds have proven antioxidant activity and anti-cancer properties. The activity of LAB during the fermentation is not only lactic acid production but also several enzymatic activities such as proteolysis, which lead to an increase in the content of free amino acids in the samples and an increase in free polyphenolics and flavonoids [36,37]. The TFCs obtained for both samples were lower than those detected in the cited studies, but it should be noted that the study presented here used BCPC, which is formed after the cold-pressing process and may have contained lower amounts of flavonoids (with particular emphasis on fat-soluble ones) than raw black cumin. It should be remembered that comparing the total polyphenol and flavonoid content determined in various studies (different standards) and converted with different formulae is subject to error [38]. 

### 3.4. Color Changes

Analyzing the results presented in Table 3, it could be concluded that the samples with the addition of 20% of BCPC were characterized by a higher value of the L* parameter than the samples with 30% of BCP, but after 28 days the samples tested were characterized by the same lightness (BCPC-20%—19.52 ± 0.02 and BCPC-30%—19.52 ± 0.03). The differences between samples on day 28 were not statistically significant (*p* > 0.05). The initial differences in the lightness might be caused by a different ratio of BCPC in the samples. Additionally, the fermentation process influenced other parameters of color (*p* < 0.05); notably, the L* was increased in both samples on day 5 (*p* < 0.05). Analyzing redness (a*) and yellowness (b*) of the tested samples, irrespective of the percentage of BCPC, there was a decrease in the a* and b* color coordinates in both cases. The reason for this phenomenon can be attributed to the properties of fermented BCPC. The process of fermentation may affect the pigments contained in BCPC, causing changes in the final color of the product, as has been seen in other studies using flaxseed press cake [5]. 

### 3.5. Rheological and Textural Changes 

As presented in Table 4, it could be observed that both samples exhibited a shear-thinning behavior during the storage time (*n* < 1.0). These findings agree with results obtained by Abu-Jdayil [12] for black cumin paste produced from whole seeds. The author suggested also that this mechanism is caused by disruption of the three-dimensional structure through the breaking of primary and secondary bonds and that the yield stress is evidence of the presence of the crystal network. The increase of the yield stress (τ_y_) is linked with the stability of the sample because is expressed as the moment when the system starts to flow [39]. The kefir-like beverages showed an increase in yield stress during storage, which was also reported for traditional dairy kefir fortified with whey protein concentrate (WPC) [13]. According to Abu-Jdayil [12], the black cumin paste is a very stable product, resistant to temperature stress and shearing, and the same effects were observed in the present study [11]. The increase of yield stress is correlated with changes in the K parameter. According to Sun and Gunasekaran [40], the increase of k is affected by the droplet integration and higher stability of the sample. BCPC also contains a small amount of residual oil and is a mixture of proteins and polysaccharides, as mentioned earlier. According to Abu-Jdayil [12], black cumin paste could be interpreted as 3-D fat-polymer structure, showing flexibility and resilience. These systems are often aligned with the deforming forces rather than breaking down. Additionally, in the present study, during the fermentation process, the microbial exopolysaccharide (EPS) extracted from kefir grains called kefiran could be formed [3,18]. This polymer is strongly sensitive to the shear forces, which could be illustrated as changes in the values of the parameters obtained in the Hershel–Bulkley model [23]. Generally, gels with the kefiran exhibited high yield stress, which indicates its role in the protein complexes and making the gel stronger. Changes in viscosity could also be observed during storage. In the case of BCPC-30%, the obtained values were significantly higher than for sample BCPC-20% (*p* < 0.05). The highest viscosity was observed on day 5 for BCPC-20% and on day 7 for BCPC-30%. This parameter significantly increased after the fermentation (*p* < 0.05). A similar tendency was previously described in the recent study about kefir-like beverages based on the flaxseed press cake, but obtained viscosities were higher than reported [3]. 

**Figure 2:**
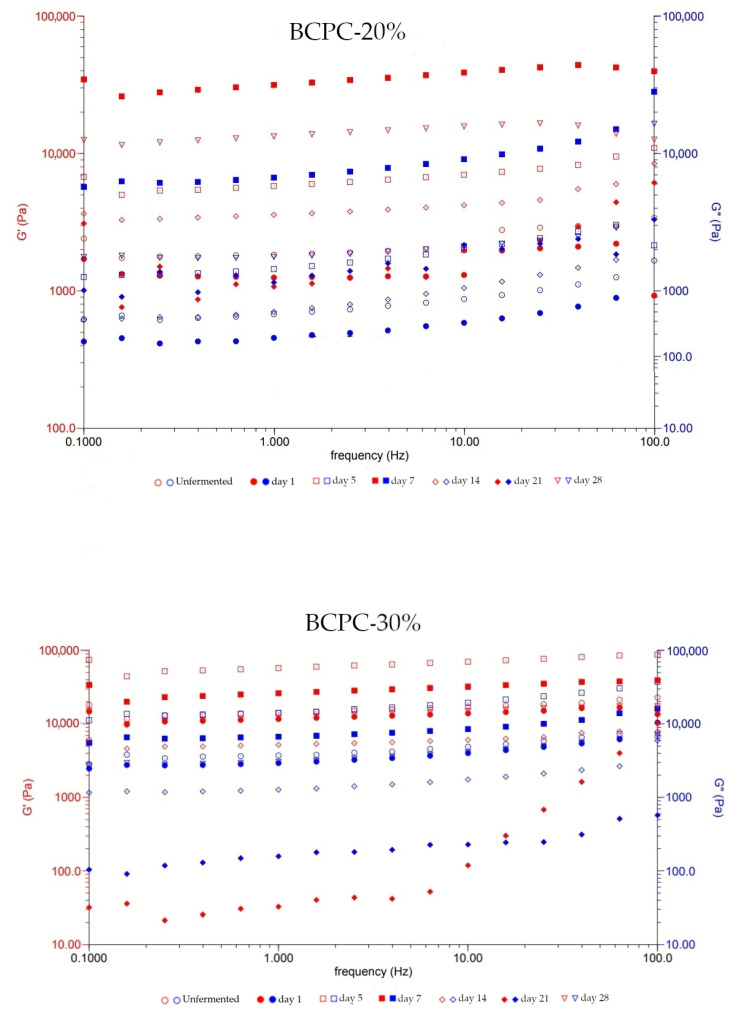
The changes of G’ (storage modulus) and G’’ (loss modulus).

A strain sweep test was used for describing the pseudoplastic behavior of the samples. This test showed a critical point at which G’ begin to decrease and the sample structure was breaking. This point is expressed as critical strain (y_c_). According to Tan et al. [41], a high level of the y_c_ indicated a stable and strong gel structure. It could be observed that the value of this point changed during the storage time. The highest y_c_ was observed on days 14 and 21. The higher elasticity could be influenced by interactions between negatively charged polysaccharides and positively charged proteins [42]. The BCPC is rich in both compounds and, additionally, during the fermentation, several interactions between these compounds might occur. The results of the changes in G’ and G” modulus also indicated the pseudoplastic behavior of the samples. The oscillatory test expressed that both moduli increased under the increased oscillatory frequency (Figure 2). Only sample BCPC-30% on day 21 exhibited higher G” than G’ under the low frequency. Additionally, this sample represented the highest critical strain point during storage. Although the increasing gel stability exhibited changes in rheological parameters, the obtained kefir-like beverages exhibited the typical rheological properties described for dairy products [43]. 

The textural changes during storage are presented in Table 5. The hardness, chewiness, and gumminess increased in both samples after fermentation (*p* < 0.05). The chewiness and gumminess decreased after 7 days of storage (*p* < 0.05). On days 0 and 1, the springiness was similar for both samples (*p* > 0.05). The results for hardness were lower than reported for traditional milk-based kefir [44,45]. However, the gumminess for sample BCPC-30% till the 7 days of storage was similar to that for traditional kefir fortified with WPC [45]. The obtained parameter, linked with the rheological effects, gives an overall impression of the diary structure. The increase of viscosity and hardness could be also connected with the production of the kefiran [3]. 

## 4. Conclusions

It was shown that BCPC fermented with kefir grains can be a good alternative to traditional kefir, especially considering the high content of microorganisms. The results indicate that BCPC beverages can provide a suitable environment for the growth of microorganisms, notably LAB and yeast. In both samples, microbial counts did not decrease below levels recommended for conventional milk–based kefir. The widely reported antimicrobial effect of black cumin oil that remained in the BCPC after pressing was also not observed. Furthermore, BCPC–based kefir-like beverages exhibited not only antioxidant properties but also interesting textural characteristics. Notably, the results obtained in rheological and textural measurements indicated the possibility of obtaining a structure similar to the well-known structure of traditional milk-based kefir. The presented results indicate that the valorization of BCPC towards new dairy alternatives may be a promising route.

## Figures and Tables

**Table 1:** The results of changes of pH, Reducing Sugar Content (RSC), and Total Solids Content (TSC) during storage.

Time of Storage (Days)							
	Unfermented	1	5	7	14	21	28
pH							
BCPC-20%	5.93 ± 0.02 ^Aa^	5.60 ± 0.01 ^Ba^	5.85 ± 0.01 ^Ca^	5.62 ± 0.04 ^Da^	5.73 ± 0.07 ^Ea^	5.76 ± 0.01 ^Fa^	5.73 ± 0.03 ^Ga^
BCPC-30%	5.89 ± 0.01^Ab^	5.74 ± 0.03 ^Bb^	5.88 ± 0.01 ^Ab^	5.78 ± 0.01 ^Cb^	5.86 ± 0.01 ^Db^	5.61 ± 0.02 ^Eb^	5.76 ± 0.01 ^Fa^
RSC (mg /mL)							
BCPC-20%	69.19 ± 1.33 ^Aa^	31.94 ± 0.00 ^Ba^	30.53 ± 0.67 ^BCa^	27.37 ± 0.38 ^Ca^	29.45 ± 0.09 ^BCa^	22.19 ± 0.09 ^Da^	29.11 ± 0.38 ^BCa^
BCPC-30%	83.85 ± 7.42 ^Ab^	31.20 ± 0.28 ^BCa^	27.97 ± 0.09 ^BCa^	27.30 ± 0.66 ^Ca^	27.16 ± 0.66 ^Ca^	21.92 ± 0.47 ^Da^	18.49 ± 0.19 ^Db^
TSC (%)							
BCPC-20%	19.90 ± 0.72 ^Aa^	15.66 ± 4.94 ^Ba^	18.72 ± 0.14 ^BCa^	18.52 ± 0.08 ^BCa^	18.14 ± 0.20 ^BCa^	19.40 ± 0.11 ^Ca^	19.63 ± 2.69 ^Ca^
BCPC-30%	28.50 ± 0.76 ^Ab^	27.98 ± 0.56 ^Bb^	26.71 ± 0.07 ^BCb^	26.39 ± 0.06 ^BCb^	24.98 ± 0.94 ^BCb^	26.57 ± 0.22 ^BCb^	23.68 ± 4.62 ^Cb^

BCPC-20%—sample with 20% (*w*/*w*) black cumin oil press cake content; BCPC-30%—sample with 30% (*w*/*w*) black cumin oil press cake content. Values are means ± standard deviation of triplicate determinations. Means with different lowercase in the same column are significantly different at *p* < 0.05. Means with different uppercase in the same row are significantly different at *p* < 0.05.

**Table 2:** Total free amino acid level (TFAAL), total polyphenolics content (TPC), and total flavonoids content (TFC).

Time of Storage (Days)							
	Unfermented	1	5	7	14	21	28
TFAAL (mg Gly/mL)							
BCPC-20%	1.19 ± 0.05 ^ABCa^	1.21 ± 0.02 ^ABa^	1.01 ± 0.06 ^Da^	0.73 ± 0.01 ^Ea^	1.16 ± 0.05 ^BCa^	1.23 ± 0.06 ^Aa^	0.94 ± 0.02 ^Fa^
BCPC-30%	1.03 ± 0.03 ^Ab^	1.00 ± 0.01 ^Ab^	0.81 ± 0.03 ^Bb^	1.05 ± 0.04 ^Ab^	1.22 ± 0.01 ^Ca^	1.21 ± 0.00 ^Db^	1.43 ± 0.00 ^Eb^
TPC (mg GAE/mL)							
BCPC-20%	6.10 ± 0.42 ^ABa^	6.25 ± 1.20 ^ABa^	6.48 ± 0.82 ^ABa^	5.53 ± 0.85 ^Aa^	6.82 ± 0.66 ^ABa^	6.88 ± 0.76 ^BCa^	6.69 ± 0.39 ^ABa^
BCPC-30%	5.96 ± 0.73 ^ABb^	6.15 ± 0.49 ^ABa^	6.22 ± 0.03 ^ABa^	6.53 ± 0.11 ^ABa^	6.46 ± 0.14 ^ABa^	5.54 ± 0.00 ^Ab^	8.15 ± 0.00 ^Cb^
TFC (mg QE/mL)							
BCPC-20%	0.35 ± 0.07 ^ABa^	1.01 ± 0.22 ^DEa^	0.69 ± 0.04 ^ABCDa^	0.31 ± 0.60 ^Aa^	0.65 ± 0.06 ^ABCDa^	0.31 ± 0.02 ^Aa^	0.54 ± 0.06 ^ABCa^
BCPC-30%	0.85 ± 0.56 ^ABb^	0.86 ± 0.21 ^ABa^	0.72 ± 0.0 ^ABCDa^	1.02 ± 0.09 ^AEb^	1.38 ± 0.15 ^Eb^	0.75 ± 0.15 ^ABCb^	0.75 ± 0.22 ^ABCa^

BCPC-20%—sample with 20% (*w*/*w*) black cumin oil press cake content; BCPC-30%—sample with 30% (*w*/*w*) black cumin oil press cake content. Values are means ± standard deviation of triplicate determinations. Means with different lowercase in the same column are significantly different at *p* < 0.05. Means with different uppercase in the same row are significantly different at *p* < 0.05.

**Table 3:** Color values of fermented beverages and unfermented (control) samples.

Time of Storage (Days)							
	Unfermented	1	5	7	14	21	28
L*							
BCPC-20%	21.29 ± 0.12 ^Aa^	20.45 ± 0.07 ^Ba^	22.00 ± 0.07 ^Ca^	22.19 ± 0.07 ^Da^	20.31 ± 0.02 ^Ea^	22.07 ± 0.01 ^Fa^	19.52 ± 0.02 ^Gb^
BCPC-30%	17.87 ± 0.02 ^Ab^	17.72 ± 0.05 ^Bb^	19.65 ± 0.02 ^Cb^	18.89 ± 0.11 ^Db^	17.78 ± 0.03 ^Eb^	19.72 ± 0.04 ^Fb^	19.52 ± 0.03 ^Eb^
a*							
BCPC-20%	2.18 ± 0.03 ^Aa^	2.42 ± 0.03 ^Ba^	2.45 ± 0.03 ^Ca^	2.45 ± 0.03 ^Ca^	2.41 ± 0.03 ^Ba^	2.40 ± 0.03 ^Ba^	1.95 ± 0.05 ^Da^
BCPC-30%	1.87 ± 0.02 ^Ab^	2.05 ± 0.06 ^Bb^	1.96 ± 0.03 ^Cb^	1.89 ± 0.02 ^Ab^	1.76 ± 0.05 ^Db^	1.97 ± 0.05 ^Cb^	1.52 ± 0.02 ^Eb^
b*							
BCPC-20%	5.92 ± 0.06 ^Aa^	7.63 ± 0.04 ^Ba^	8.08 ± 0.04 ^Ca^	8.09 ± 0.05 ^Ca^	7.17 ± 0.05 ^Da^	7.58 ± 0.03 ^Ea^	5.37 ± 0.08 ^Fa^
BCPC-30%	4.53 ± 0.02 ^Ab^	5.31 ± 0.02 ^Bb^	5.23 ± 0.07 ^Cb^	4.82 ± 0.04 ^Db^	3.79 ± 0.04 ^Eb^	5.04 ± 0.03 ^Fb^	3.28 ± 0.03 ^Gb^

BCPC-20%—sample with 20% (*w*/*w*) black cumin oil press cake content; BCPC-30%—sample with 30% (*w*/*w*) black cumin oil press cake content. Values are means ± standard deviation of triplicate determinations. Means with different lowercase in the same column are significantly different at *p* < 0.05. Means with different uppercase in the same row are significantly different at *p* < 0.05.

**Table 4:** Changes of Hershel–Bulkley parameters, viscosity, and critical point during the storage time.

Sample *		Time of Storage (Days)					
	Unfermented	1	5	7	14	21	28
	n (-)						
BCPC-20%	0.60 ± 0.01 ^Aa^	0.95 ± 0.00 ^Ba^	0.91 ± 0.01 ^Ca^	0.73 ± 0.04 ^Da^	0.86 ± 0.01 ^Ea^	0.61 ± 0.03 ^Fa^	0.74 ± 0.00 ^Da^
BCPC-30%	0.58 ± 0.01 ^Aa^	0.97 ± 0.02 ^Bb^	0.91 ± 0.00 ^Ca^	0.79 ± 0.00 ^Db^	0.83 ± 0.01 ^Eb^	0.60 ± 0.01 ^Fa^	0.65 ± 0.00 ^Gb^
	K (Pa·s^n^)						
BCPC-20%	1.00 ± 0.02 ^Aa^	3.33 ± 0.10 ^Ba^	3.89 ± 0.05 ^Ca^	4.02 ± 0.02 ^Da^	4.85 ± 0.00 ^Ea^	4.83 ± 0.02 ^Ea^	8.05 ± 0.04 ^Fa^
BCPC-30%	1.08 ± 0.00 ^Aa^	4.15 ± 0.09 ^Bb^	6.72 ± 0.04 ^Cb^	5.12 ± 0.02 ^Db^	4.54 ± 0.01 ^Eb^	4.19 ± 0.02 ^Bb^	4.21 ± 0.02 ^Bb^
	τ_y_ (Pa)						
BCPC-20%	5.68 ± 0.11 ^Aa^	3.03 ± 0.05 ^Ba^	3.14 ± 0.01 ^Ca^	3.74 ± 0.01 ^Da^	6.59 ± 0.03 ^Ea^	9.37 ± 0.01 ^Fa^	9.64 ± 0.04 ^Ga^
BCPC-30%	3.17 ± 0.09 ^Ab^	4.78 ± 0.01 ^Bb^	4.40 ± 0.05 ^Cb^	3.41 ± 0.03 ^Db^	2.79 ± 0.02 ^Eb^	3.01 ± 0.02 ^Fb^	9.00 ± 0.01 ^Gb^
	Viscosity (Pa·s)						
BCPC-20%	160,000 ± 0.20 ^Aa^	13650 ± 0.54 ^Ba^	92260 ± 0.21 ^Ca^	8782 ± 0.15 ^Da^	8043 ± 0.09 ^Ea^	2407 ± 0.10 ^Fa^	56.72 ± 0.12 ^Ga^
BCPC-30%	659,000 ± 1.23 ^Ab^	970700 ± 0.34 ^Bb^	2111000 ± 1.12 ^Cb^	7987100 ± 0.43 ^Db^	103100 ± 0.45 ^Eb^	109.70 ± 0.21 ^Fb^	64.97 ± 0.52 ^Gb^
	y_c_ (%)						
BCPC-20%	60.26 ± 0.20 ^Aa^	16.68 ± 0.05 ^Ba^	22.71 ± 0.03 ^Ca^	28.64 ± 0.09 ^Da^	76.05 ± 0.12 ^Ea^	63.08 ± 0.02 ^Fa^	39.43 ± 0.11 ^Ga^
BCPC-30%	18.85 ± 0.14 ^Ab^	24.16 ± 0.10 ^Bb^	16.06 ± 0.08 ^Cb^	17.67 ± 0.02 ^Db^	64.31 ± 0.05 ^Eb^	107.75 ± 0.15 ^Fb^	21.91 ± 0.08 ^Gb^

BCPC-20% *—sample with 20% (*w*/*w*) black cumin oil press cake content; BCPC-30% *—sample with 30% (*w*/*w*) black cumin oil press cake content. Values are means ± standard deviation of triplicate determinations. Means with different lowercase in the same column are significantly different at *p* < 0.05. Means with different uppercase in the same row are significantly different at *p* < 0.05.

**Table 5:** Changes of the textural parameters during storage.

Sample *		Time of Storage (Days)					
	Unfermented	1	5	7	14	21	28
	Hardness (N)						
BCPC-20%	0.04 ± 0.00 ^Aa^	0.03 ± 0.00 ^Aa^	0.09 ± 0.00 ^Ba^	0.15 ± 0.02 ^Ca^	0.10 ± 0.00 ^Ba^	0.27 ± 0.01 ^Da^	0.36 ± 0.00 ^Ea^
BCPC-30%	0.04 ± 0.00 ^Aa^	0.05 ± 0.01 ^Ab^	0.19 ± 0.00 ^Bb^	0.25 ± 0.00 ^Cb^	0.27 ± 0.01 ^Db^	0.50 ± 0.01 ^Eb^	0.25 ± 0.02 ^CDb^
	Springiness (N)						
BCPC-20%	0.48 ± 0.04 ^Aa^	0.61 ± 0.02 ^Bb^	0.57 ± 0.02 ^Ca^	0.81 ± 0.02 ^Da^	0.69 ± 0.03 ^Ea^	0.79 ± 0.01 ^Da^	0.60 ± 0.02 ^Ba^
BCPC-30%	0.78 ± 0.02 ^Aa^	0.74 ± 0.02 ^Bb^	0.50 ± 0.06 ^Cb^	0.73 ± 0.01 ^Bb^	0.53 ± 0.03 ^Db^	0.80 ± 0.02 ^Aa^	0.59 ± 0.02 ^Ea^
	Gumminess (N)						
BCPC-20%	0.02 ± 0.00 ^Aa^	0.02 ± 0.01 ^Aa^	0.03 ± 0.01 ^ABa^	0.04 ± 0.01 ^Ca^	0.03 ± 0.01 ^BCPCa^	0.08 ± 0.00 ^Da^	0.10 ± 0.00 ^Ea^
BCPC-30%	0.24 ± 0.01 ^Ab^	0.18 ± 0.03 ^Bb^	0.20 ± 0.00 ^BCPCb^	0.26 ± 0.00 ^Db^	0.21 ± 0.00 ^Cb^	0.24 ± 0.01 ^Ab^	0.43 ± 0.01 ^Eb^
	Chewiness (N)						
BCPC-20%	0.01 ± 0.00 ^Aa^	0.01 ± 0.01 ^Aa^	0.02 ± 0.01 ^Aba^	0.03 ± 0.00 ^Ba^	0.02 ± 0.00 ^Aa^	0.07 ± 0.01 ^Ca^	0.06 ± 0.00 ^Ca^
BCPC-30%	0.20 ± 0.00 ^Ab^	0.13 ± 0.00 ^Bb^	0.10 ± 0.00 ^Cb^	0.19 ± 0.00 ^Ab^	0.14 ± 0.01 ^Db^	0.19 ± 0.02 ^Ab^	0.25 ± 0.06 ^Eb^

BCPC-20% *—sample with 20% (*w*/*w*) black cumin oil press cake content; BCPC-30% *—sample with 30% (*w*/*w*) black cumin oil press cake content. Values are means ± standard deviation of triplicate determinations. Means with different lowercase in the same column are significantly different at *p* < 0.05. Means with different uppercase in the same row are significantly different at *p* < 0.05.

## Data Availability

The data presented in this study are available on request from the corresponding author.
